# Author Correction: Non-coding RNAs participate in the regulatory network of CLDN4 via ceRNA mediated miRNA evasion

**DOI:** 10.1038/s41467-021-23211-y

**Published:** 2021-05-19

**Authors:** Yong-xi Song, Jing-xu Sun, Jun-hua Zhao, Yu-chong Yang, Jin-xin Shi, Zhong-hua Wu, Xiao-wan Chen, Peng Gao, Zhi-feng Miao, Zhen-ning Wang

**Affiliations:** 1 Department of Surgical Oncology and General Surgery, The First Affiliated Hospital of China Medical University, 155 North Nanjing Street, Heping District, Shenyang City, 110001 China; 2 Division of Gastroenterology, Department of Medicine, Washington University School of Medicine, St Louis, MO 63110 USA

Correction to: *Nature Communications* 10.1038/s41467-017-00304-1, published online 18 August 2017

In the original version of this Article, the images in the Figure 2c column labelled “CLDN4+ miR-3620-3p mut’ were inadvertently replaced with duplicates from the Figure 2c column labelled “CLDN4+”. In addition, the Figure 2f column “CLDN4+ miR-3620-3p mut’ was inadvertently labelled “CLDN4+ miR-3620-3p”. The original and the corrected figures are presented below.





