# Supplementary material for: Non-coding RNAs participate in the regulatory network of CLDN4 via ceRNA mediated miRNA evasion
Source: Nat Commun. 2017 Aug 18;8:289. doi: 10.1038/s41467-017-00304-1 (PMC5561086; doi:10.1038/s41467-017-00304-1)
Supplement: Supplementary file 1 — Supplementary Information [file 41467_2017_304_MOESM1_ESM.pdf]

File name: Supplementary Information
Description: Supplementary figures, supplementary tables, supplementary methods and supplementary references.

File name: Supplementary Data 1
Description: miRNA Expression Profiling Data

File name: Supplementary Data 2
Description: LncRNA Expression Profiling Data

File name: Supplementary Data 3
Description: mRNA Expression Profiling Data

File name: Supplementary Data 4
Description: Differentially expressed miRNAs between gastric cancer and non-tumorous adjacent tissues

File name: Supplementary Data 5
Description: Differentially expressed lncRNAs between gastric cancer and non-tumorous adjacent tissues

File name: Supplementary Data 6
Description: Differentially expressed mRNAs between gastric cancer and non-tumorous adjacent tissues

File name: Supplementary Data 7
Description: List of miRNA and their predicted targets in the intersection of our predicted result and three miRNA prediction programs

File name: Supplementary Data 8
Description: List of predicted pathways based on the differentially expressed genes using KEGG pathways

File name: Supplementary Data 9
Description: The expression of RNAs based on RNA-sequencing

File name: Supplementary Data 10
Description: Clinical information of 104 patients with gastric cancer

File name: Supplementary Data 11
Description: Sequences of primers used in reverse transcription and real-time PCR

File name: Peer review file
Description:

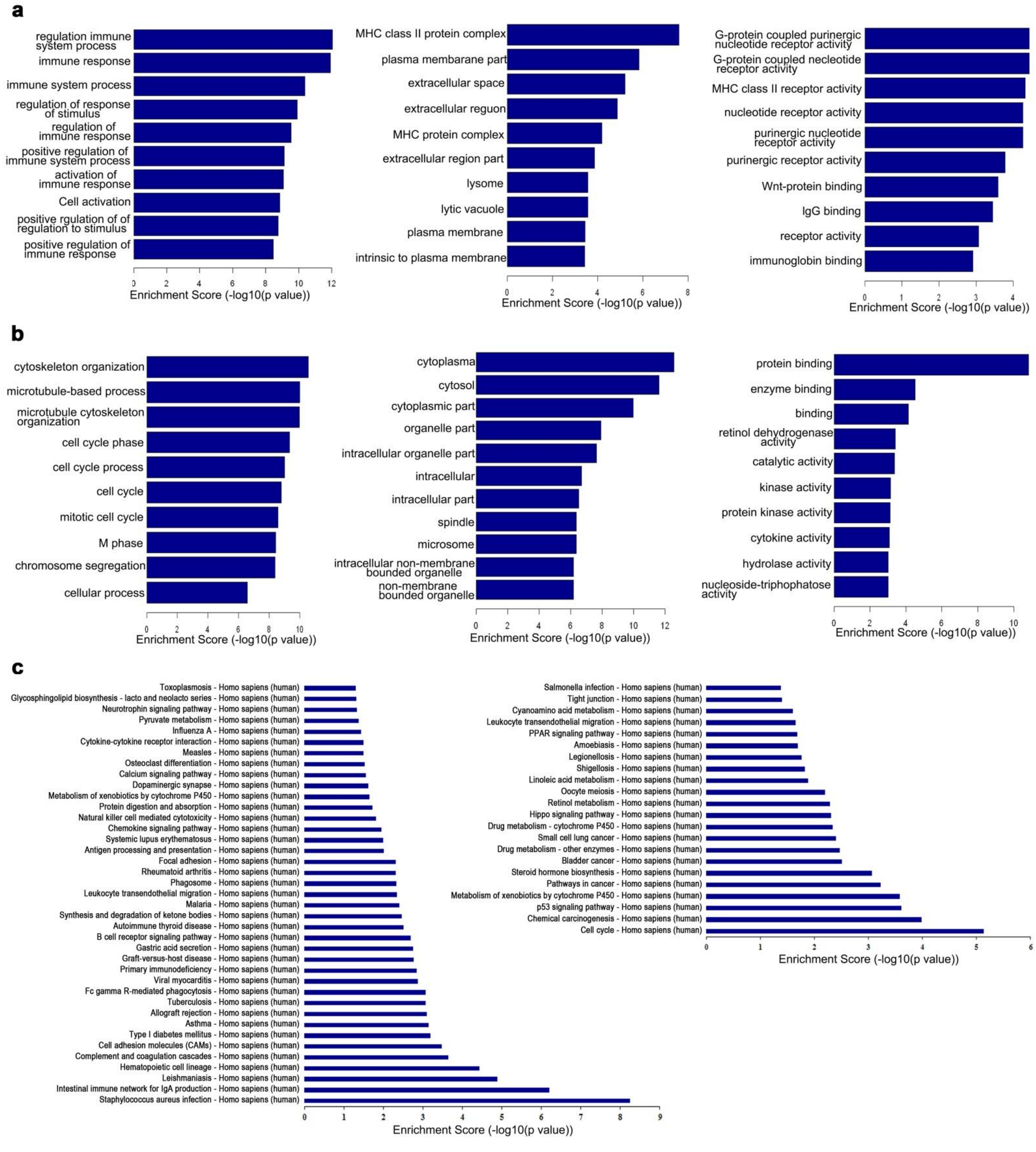

Supplementary Figure 1. Results of GO analysis and pathway analysis of the gene products

(a) The top ten Enrichment Score values of the significantly down-regulated terms for Biological Process, Cellular Components, and Molecular Function. **(b)**The top ten Enrichment Score values of the significantly up-regulated terms for Biological Process, Cellular Components, and Molecular Function. **(c)**The Enrichment score (-log10 (P value)) values of all significantly down-regulated and up-regulated pathways.

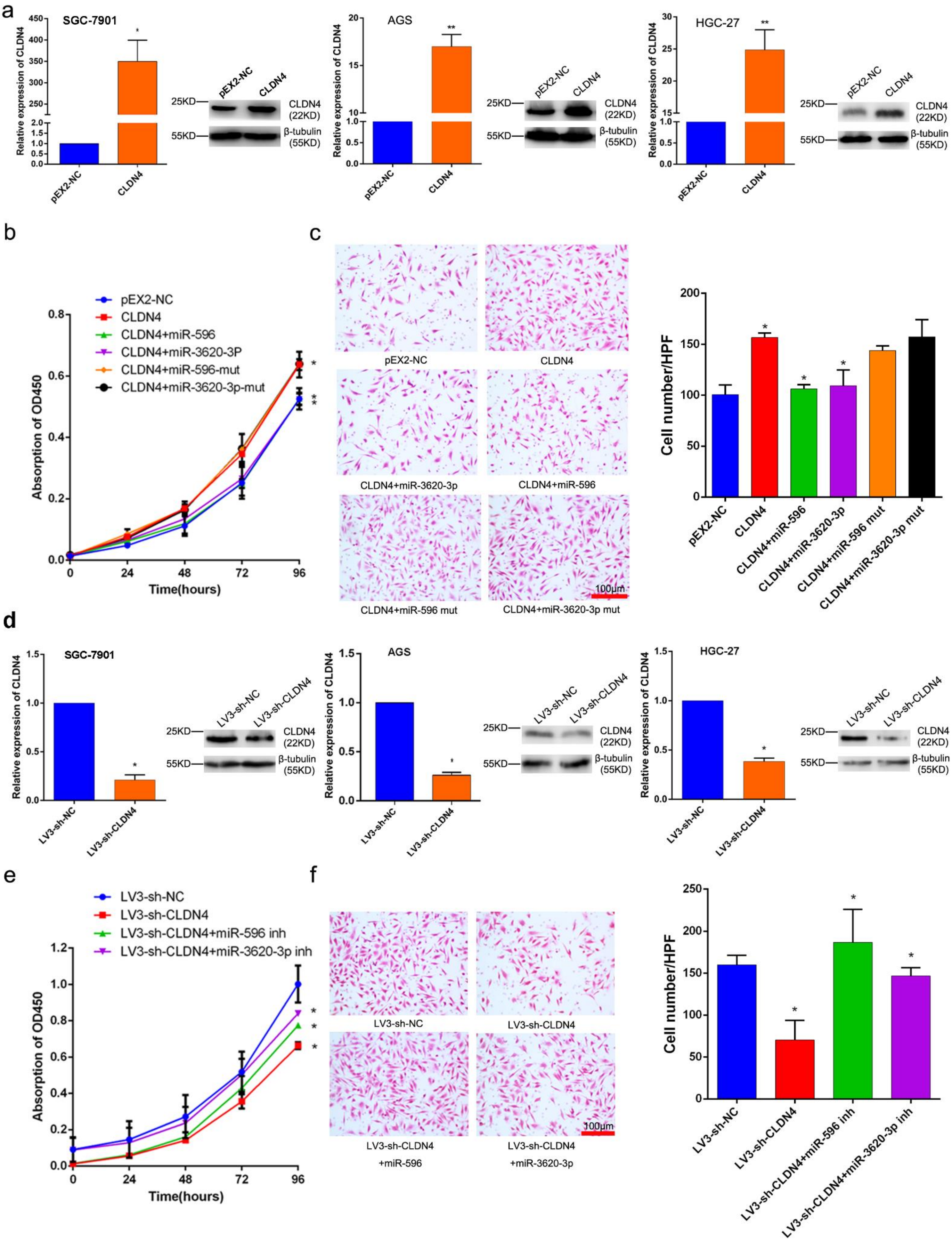
a
SGC-7901
AGS
HGC-27
Relative expression of CLDN4
pEX2-NC
CLDN4
25KD
55KD
CLDN4 (22KD)
β-tubulin (55KD)
b
pEX2-NC
CLDN4
CLDN4+miR-596
CLDN4+miR-3620-3P
CLDN4+miR-596-mut
CLDN4+miR-3620-3p-mut
Absorption of OD450
Time(hours)
c
pEX2-NC
CLDN4
CLDN4+miR-3620-3p
CLDN4+miR-596
CLDN4+miR-596 mut
CLDN4+miR-3620-3p mut
100μm
Cell number/HPF
CLDN4+miR-3620-3p
CLDN4+miR-596 mut
d
SGC-7901
AGS
HGC-27
LV3-sh-NC
LV3-sh-CLDN4
e
LV3-sh-NC
LV3-sh-CLDN4
LV3-sh-CLDN4+miR-596 inh
LV3-sh-CLDN4+miR-3620-3p inh
Absorption of OD450
Time(hours)
f
LV3-sh-NC
LV3-sh-CLDN4
LV3-sh-CLDN4 +miR-596
LV3-sh-CLDN4 +miR-3620-3p
100μm
Cell number/HPF

Supplementary Figure 2. The transfection efficiency and function of CLDN4 in GC cells

(a) The transcriptional and translational levels of CLDN4 in pEX2-NC and CLDN4 cells. **(b)** Cell proliferation was assessed daily for four days using the Cell Counting Kit-8 (CCK-8) assay in CLDN4 overexpressing HGC-27 cells. **(c)** Transwell assays were used to evaluate the involvement of CLDN4 for invasion in CLDN4 overexpressing HGC-27 cells. Cells were incubated for 24h and counted under the microscope. Original magnification×200. Scale bars=100μm. **(d)** The transcriptional and translational levels of CLDN4 in LV3-sh-NC and LV3-sh-CLDN4 cells. **(e)** Cell proliferation was assessed daily for four days using the CCK-8 assay in CLDN4 knockdown HGC-27 cells. **(f)** Transwell assays were used to evaluate the involvement of CLDN4 for invasion in CLDN4 knockdown HGC-27 cells. Cells were incubated for 24h and counted under the microscope. Original magnification×200. Scale bars=100μm. Data are shown as mean±s.d., n=3. The data statistical significance is assessed by Student's t-test. *represents $P<0.05$.

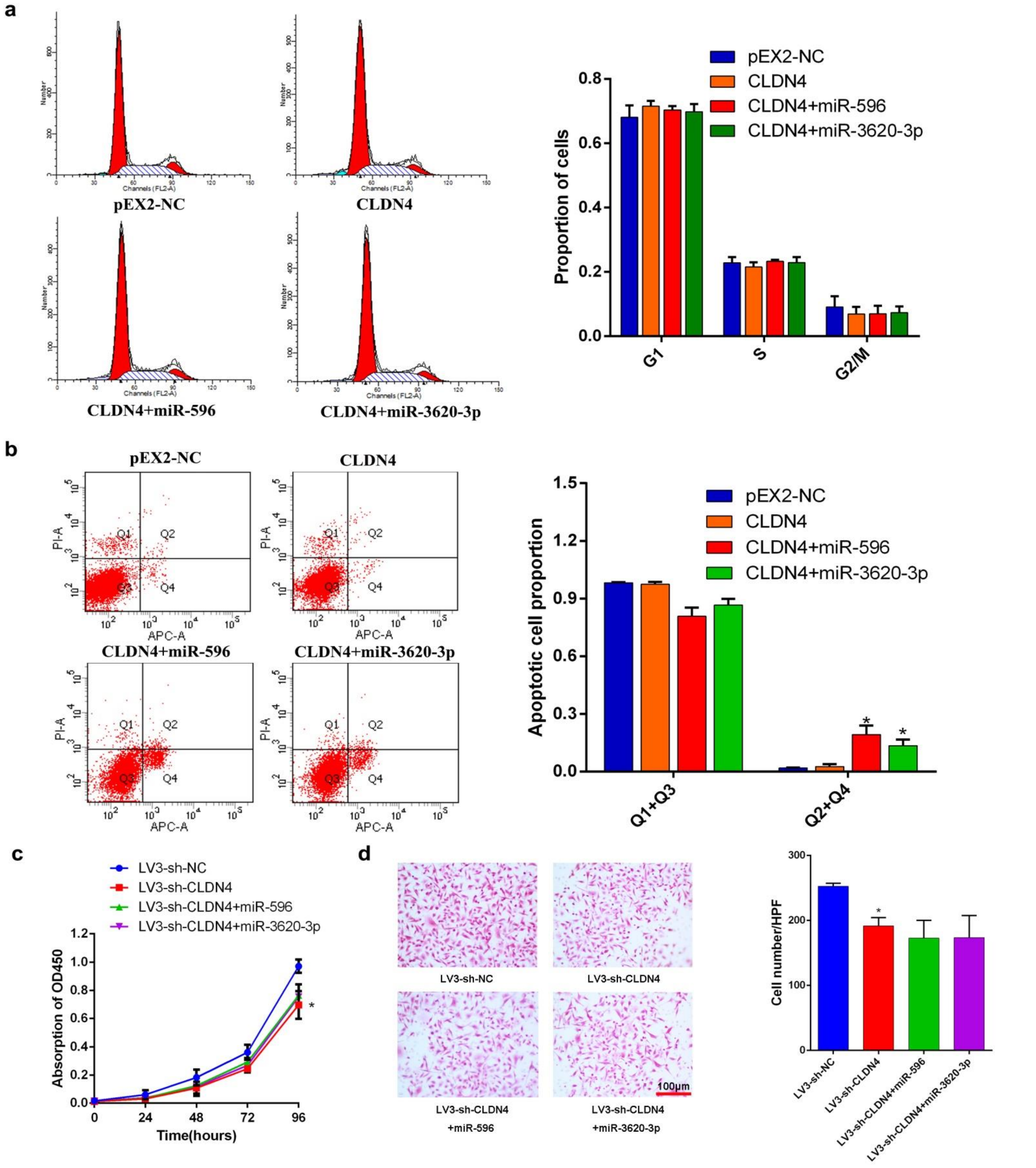

Supplementary Figure 3. The results of cell-cycle and apoptosis analysis

(a) FACS analysis showed no significant changes in the G1, S and G2/M phases between pEX2-NC cells, CLDN4 overexpressing cells, CLDN4 overexpressing cells transfected with miR-596, and CLDN4 overexpressing cells transfected with miR-3620-3p. **(b)** Cells were stained with both Annexin V-APC and PI. The cells which were positive for Annexin V-APC were counted as apoptotic cells. The data were presented as the proportion of apoptotic cells. **(c)** Cell proliferation was assessed daily for four days using the CCK-8

assay in CLDN4 knockdown AGS cells. **(d)** Transwell assays were used to evaluate the involvement of CLDN4 for invasion in CLDN4 knockdown AGS cells. Cells were incubated for 24h and counted under the microscope. Original magnification×200. Scale bars=100μm. Data are shown as mean±s.d.. The data statistical significance is assessed by Student's t-test. *represents the $P<0.05$.

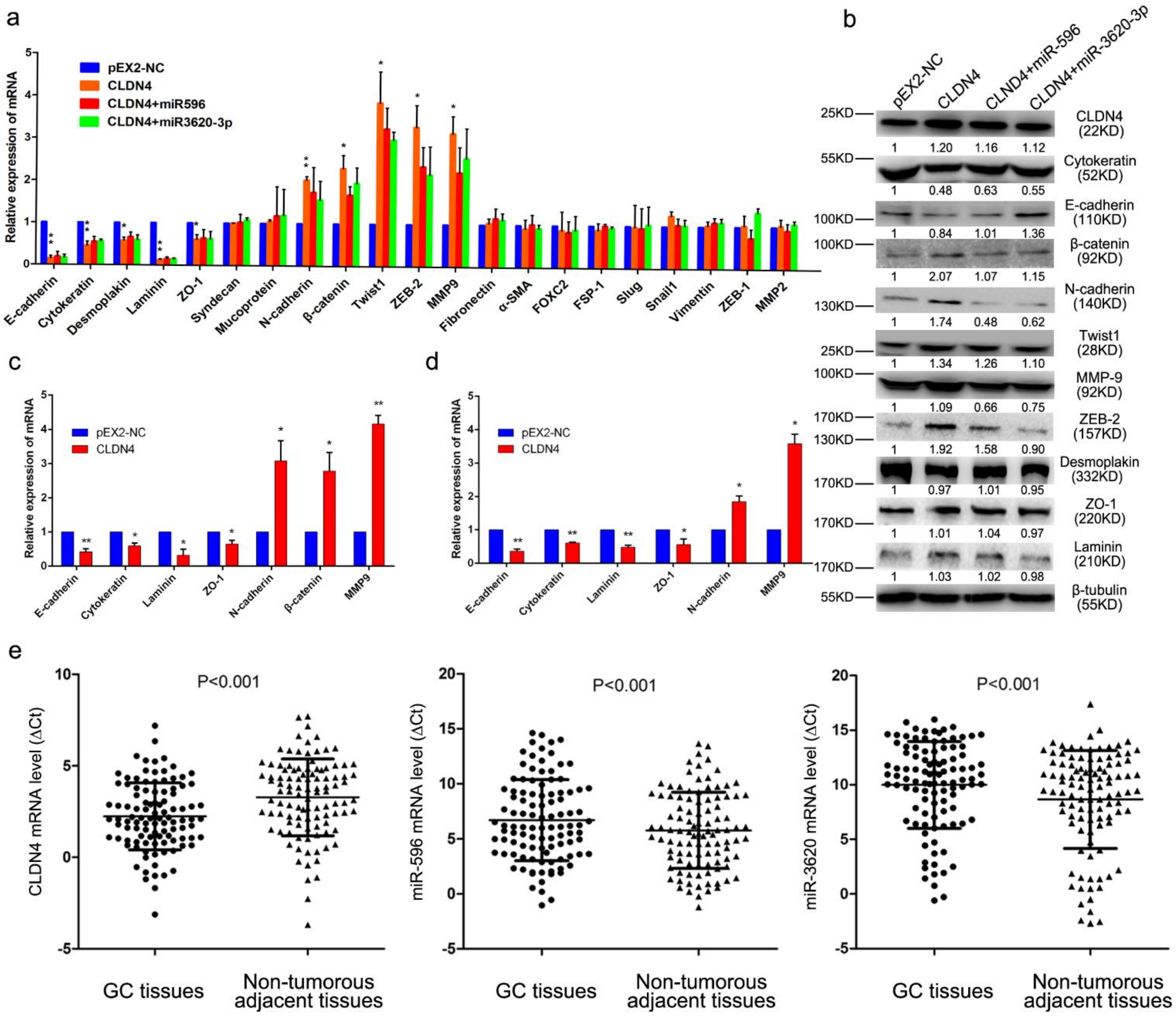

Supplementary Figure 4. The expression of EMT markers in vitro and CLDN4, miR-596, miR-3620-3p *in vivo*

(a) The transcriptional levels of EMT markers CLDN4 overexpressing SGC-7901 cells. **(b)** The translation levels of EMT markers in CLDN4 overexpressing SGC-7901 cells. **(c)** The transcriptional levels of EMT markers CLDN4 overexpressing AGS cells. **(d)** The transcriptional levels of EMT markers CLDN4 overexpressing HGC-27 cells. **(e)** The expression levels of CLDN4, miR-596, miR-3620-3p in human GC tissues and matched non-tumorous adjacent tissues were analyzed by real-time PCR. The ΔCT values (CLDN4 normalized to GAPDH, miRNAs normalized to U6 snRNA) were subjected to the Wilcoxon signed-rank test. Larger ΔCT value indicated lower expression. Data are shown as mean±s.d., n=3. *represents P<0.05, **represents P<0.01.

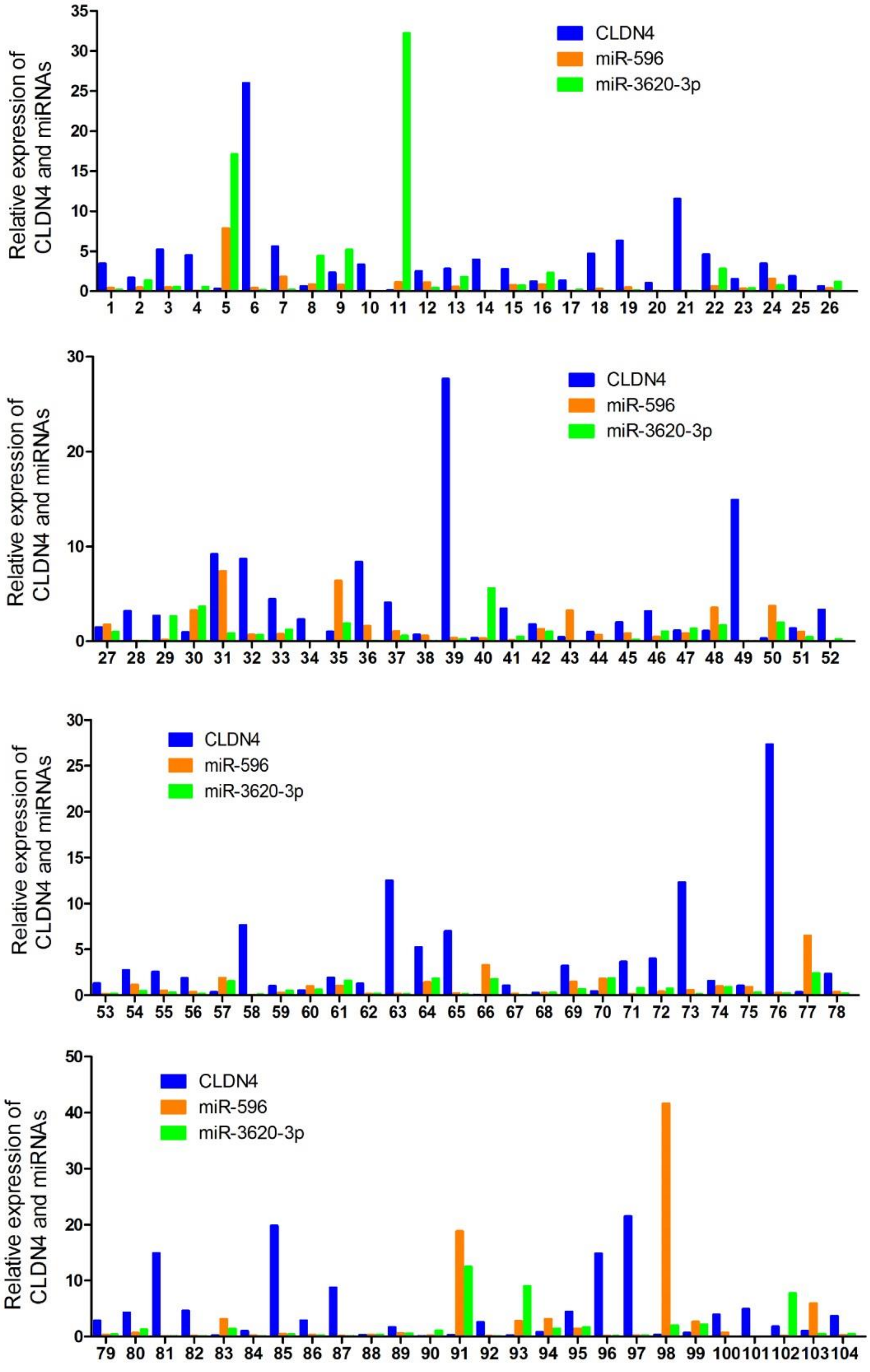

Supplementary Figure 5. The expression of CLDN4, miR-596 and miR-3620-3p in the whole 104 patients

a

1 AGACATTTCT AAAAGGCCCC CTCCCCGCTG CCGGGTGGAG AGGGAGCGTG GGGGGATCCG CCTTCTGCCC CCGAGCCCCC AGCCAGCCTG GGCCCCCCAG
101 GGTCACCCTG GGGCTCTGAA GGGGGCTTCG GCCGCTGGTT GAATGAAGGG CCACAGAAAA TGAAGCTGGC GCAACGACCG CAGAACCCTC AGTGGGCACC
201 AGGACCACGG AGCTCAGGGT GCCAATGGTG ATGGCTGGGG GTGGGGAGTA CTGTGGGCAG CAGGGGATGC TGAGCGGGGT TTGGGGCTCC CCCTTTCCAT
301 ACTCAGTCTT CCTGCCAGCC TCAGAGCTGA GTACACCGTT GGCGATTAAT AAGCGCTTGC AGCTCCCTTC CCTGGCCAGC CTGAGGGGAG CGGCAGTGGC
401 CCGTATGGGG TGGGGGTGGC GGTGGAGTGC GTCCTGGCGC AGAGCTGGCA CACAGCACAA ACAAACGCCT TGGCGGGAGT GGGTCTGTCC CGGAAGGGAG
501 CCCAGGGGTT ATTGGGGTTG GGGCTGCGCC CCAAAGCGGG AGGGCCCTGC GAGAAGGGAG GCCCTGGCTG GAGGGGGACC CTGAGACTGG AGGTGCCGGA
601 CCCGGATGCG GGGCCAGATG GAGACAACAG GAAGGAAAGA GACCTTCCTG GTCACACTTG GGCCACAGGA GAACAGGCAG CGGCCCAGGA GGATCCAGGG
701 TCCTGATGGT GGTTGAGAAG CTGGTTCTTA GTGATCACTC AGAAACGTCG GCCTGGCCTT GTGGGGTCAG ACCTTGCATC TCAGTCAGCC CAGGGAGAAG
801 AAGAAGATGG TCCACACCCA AGTGCAGGGA ACATCGTGGC AGTCGGCTGG GTGCCTGCGT CCAGGCGAGG ACACCTCCCG CATCAGGGAA ACACACGTTT
901 CTGGAGTGAG GAGGCTGAAG GCAGGGCCCA GAGGAGAGCT GAGCCATGGA AGGAGGTGTG TGCATGGATG GTGAGCTAGA GCAGGTGCAG GTGCCTCAGG
1001 GAGGATGTGT GGGACGAACT GACTCAGGGA ACCATAAGAA ATGCTTTCAC CAAACAGGAG AAACCTGAAG GTCTGGGTCC AGAGCCTCAG ATCTTACACT
1101 GGCAGCACAC AGGGACACAA CAGTTGGACT GGCAGCAACA GGGCTTGCAG CAGCTGGACT GGCAGCACAC GGGGACACAG CAGCTGGACT GGCAGCAGCA
1201 GGGCTTGCAG CAGCTGGACT GGCAGCAGGA TGATCCACAG CCTGAGGAGC AGCAACAGGG CTTACAACAG CTGGACTGGG AGCAGCCACA AGAACCACAG
1301 CCCCCCTTGG AACCCCCACA GGAGCCACAG CCCCCCTTGG AGCCCCCACA GGAGCCACAG CCCCCCTTGG AGCCCCCACA GGAGCCACAG CTGGTGCAGG
1401 AACAGGCTGG CACCCAGGAG CACACGGGCT TGCAGCAGCA GACAGGCACA TAACATCTGG AGCCACATCC CCCACAGCTG GAGCTGCAGC CCCCACAGCC
1501 AGAGCCACAG CCCCCACGGC CGGAGCCACA GCCCCCACAG CCAGAGCCAC AACCCCCACA GCTGGAGCCA CAGCCCCCAC AGCCGGAGCC ACAGCCTCTG
1601 GAGCAGCCAC AGCAGCCCAT GGTTCTGGTG GATTGAGGGT GGAGCAGGTA GAGGAGCAGG TGAGAGGGAG GTGCAGGTGT GGAGCTCCCT GAGCCTGGGC
1701 TCTTTATATA CCTGTCCAGA TGTCAGGCAT GACACAGGGT CCCTTTCTTG TGACTGTTTA CACTATTTTT CCAGAGCTCT ATTTTTTTCC TCTTTGCTAG
1801 TGACTTCCTT CTGGCTCAGT TGAGCATCTA CTTTCTTTGT TTTCTAAATT TGTCTTTTTC CCCATTTGTT TTGGCCCCTA CAATTAAAAC CTCAGCTCCA
1901 GGCTGTCTGG TTCTTCCTGC AAAGCTCCAG GGTGCTGGTC ACCTGCTCTC TGCTGACCAC ATGTGACCAA TGGGCAACAG CCTCTGCCCA CGTGCTCTCA
2001 TCTTTCCTGT GTTGACTCCC TCAATAATAT TAATTTTACA TTTTTAGATT TCAAAATTAT CCACGATCTT TATACTCATG CCAGTCTCAG CTTTCCGGGT
2101 GTGTTAGACC ATTCTTTGCA TTGCTCTAAA GAAATACTTG AGGCTGGGTA ATTTATAAAG GAAAGAGGTT AGAATGGCTC ATGGTTCTGC AGGCTGCACA
2201 AGCATGGCAC CCACCTCTGC TCAGCTTCTG GGGAGGCCCT CAGGGAGTTT TCCTCACGGT GGAAGGCGAA GCAGGAACAG GCACACCACA TGGTGAGAGT
2301 GGGAGCAAGG GGTGAGGAGG AGCCACACAC TTGTGAACAA CCAGATCTGA GTGAACACAC TCATCGCCAA GGGGGTGGCA CTAAGTCACT CATGAGGGAT
2401 CCACCCCCAT GACCCAGACA CCTCCCTGCA GGCCCCTCCT TCAACACTGG CCAACAACTA TATGAAAAAA TGCTCCACAT CACTAACTAT TCGGGGAATG
2501 TAAATCGAAA CCACCACGAG ATACCATCTC ATATCAGTCA GAATGGCTTT TGAT

b

1 TACGCCTCGT CCTCGCCCTC CTCCTCCTCG AACTCCCCTT GTTCGTCGGC CGTGGCGTCC TGGTACTGCT GGTACTCGGA CACCAGGTCG TTCATGTTGC
101 TCTCGGCCTC GGTGAACTCC ATCTCGTCCA TGCCCTCGCC CGTGTACCAG TGCAGGAAGG CCTTGCGCCG GAACATGGCC GTGAACTGCT CGGAGATGCG
201 CTTGAACAGC TCCTGGATGG CCGTGCTGTT GCCGATGAAG GTGGCCGACA TCTTCAGGCC GCGGGGCGGG ATGTCGCACA CGGCCGTCTT CACGTTGTTG
301 GGGATCCACT CCACGAAGTA GCTGCTGTTC TTGTTCTGCA CGTTGAGCAT CTGCTCGTCC ACCTCCTTCA TGGACATGCG GCCCCGGAAG ATGGCAGCCA
401 CCGTCAGGTA GCGGCCGTGG CGCGGGTCGC AGGCGGCCAT CATGTTCTTG GAGTCGAACA TCTGCTGGGT GAGCTCGGGC ACCGTGAGCG CCCGGTACTG
501 CTGGCTGCCC CGGCTGGTCA GGGGCGCGAA GCCGGGCATG AAGAAGTGCA GGCGAGGGAA GGGCACCATG TTCACCGCCA GCTTGCGCAG GTCTGCGTTC
601 AGCTGGCCCG GGAAGCGCAG GCAGGTGGTG ACCCCGCTCA TGGTGGCCGA CACCAGGTGG TTGAGGTCCC CGTAGGTGGG GGTGGTCAGC TTCAGGGTGC
701 GGAAGCAGAT GTCATACAGG GCCTCGTTGT CAATGCAGTA GGTTTCATCT GTGTTTTCCA CCAGCTGGTG GACCGAGAGG GTGGCGTTGT AGGGCTCCAC
801 CACCGTGTCT GACACCTTGG GTGAGGGCAT GACGCTGAAG GTGTTCATGA TGCGGTCTGG GTACTCTTCC CGGATCTTGC TGATGAGCAG GGTGCCCATC
901 CCGGACCCCG TGCCGCCCCC CAGAGAGTGG GTCAGCTGGA AGCCCTGGAG ACAGTCACAG CTCTCTGACT CCTTCCTCAC CACATCCAGG ACCGAGTCGA
1001 CCAGCTCGGC TCCCTCTGTG TAGTGGCCCT TGGCCCAGTT ATTCCCGGCT CC

Supplementary Figure 6. The nucleotide sequences of lncRNA-KRTAP5-AS1 and lncRNA-TUBB2A

(a) The nucleotide sequence of full-length human lncRNA-KRTAP5-AS1, Position: hg19 chr11:1,594,477-1,620,414 Size: 2554; UCSC Genome Browser on Human Feb. 2009 (GRCh37/hg19) Assembly. **(b)** The nucleotide sequence of full-length human lncRNA-TUBB2A, Position: hg19 chr6:3,224,986-3,226,037 Size: 1052; UCSC Genome Browser on Human Feb. 2009 (GRCh37/hg19) Assembly.

a MiR-596 binding sites on lncRNA-KRTAP5-AS1

Matching	Position and MFE
1 target 5' GCCCGUAUGGGGUGGGGGUGGCG 3'	position 399
miRNA 3' GGGCU------CCUCGGCCCGUCCGAA 5'	MFE: -38.0 kcal/mol
2 target 5' UCCCGGAAGGGAGCCCAGGGGUUAUUGGGGUUGGGGCUG 3'	position 488
miRNA 3' GGGC-------UCCUCGG---------------------------CCCG------UCCGAA 5'	MFE: -35.6 kcal/mol
3 target 5' GCCUGAGGAGCA----GCAACAGGGCUUA 3'	position 1240
miRNA 3' GGGCUCCUCGGCCCG---------UCCGAA 5'	MFE: -33.8 kcal/mol
4 target 5' ACCC--AGGAGCCGGGC-ACA-AGGCUU 3'	position 1412
miRNA 3' GGGCUCCUCGGCCCG---------UCCGAA 5'	MFE: -33.3 kcal/mol
5 target 5' ACUGGAGGUGCCGGACCCGGAUGCGGGG 3'	position 586
miRNA 3' GGGCUCCUCGGCC------------------CGUCCGAA 5'	MFE: -31.5 kcal/mol

b MiR-3620-3p binding sites on lncRNA-KRTAP5-AS1

Matching	Position and MFE
1 target 5' AGGUGAGAGGGAGGUGCAGGUGUGGA 3'	position 1658
miRNA 3' GACCCACG-----CCCU----ACGUCC---CACU 5'	MFE: -33.8 kcal/mol
2 target 5' AUGGG--GUGGGG-GUGGCGGUGGA 3'	position 405
miRNA 3' GACCCACGCCCUACGUC-CCACU 5'	MFE: -33.3 kcal/mol
3 target 5' GCUGGGGGUGGGGAGUACUGUGGGCAGCAGGG-GAU 3'	position 234
miRNA 3' GACCC---------------------------ACGCCCUACGUCCCACU 5'	MFE: -33.2 kcal/mol
4 target 5' ACUGGAGGUGCCGGACCCGGAUGCGGGGC 3'	position 586
miRNA 3' GAC-----CCACGC--------------CCUACGUCCCACU 5'	MFE: -32.1 kcal/mol
5 target 5' AUGGUGAGAGUGGGA--GCAAGGGGUGAG 3'	position 2290
miRNA 3' GACC---CA----CGCCCUACG-----UCCCACU 5'	MFE: -31.7 kcal/mol

c MiR-3620-3p binding sites on lncRNA-TUBB2A

Matching	Position and MFE
1 target 5' AGGGUGCGGAAGCAGAUGUCAUACAGGGC 3'	position 694
miRNA 3' GACCCACGCC------------CUACG-----------UCCCACU 5'	MFE: -31.6 kcal/mol
2 target 5' CGGG—GCGGGAUGUC 3'	position 262
miRNA 3' GACCCACGCCCUACGUCCCACU 5'	MFE: -28.9 kcal/mol
3 target 5' UCUGGGUACUCUUCCCGGAUCUUGCUGAUGAGCAGGGUGC 3'	position 856
miRNA 3' GACCCACGC-------------CCUA-----------------------CGUCCCACC 5'	MFE: -28.7 kcal/mol
4 target 5' GCUGGCC-CGGGAAGCGCAGGCAGGUGGUGAC 3'	position 602
miRNA 3' GACCCACGCCCUA-----------CGUC-----CCACU 5'	MFE: -28.6 kcal/mol
5 target 5' CGGGUCGCAGGCGGCCAUCAUGUUCUUGGAGUCGAACAUCUGCUGGGUGAG 3'	position 423
miRNA 3' GACCCA-----------CGC-----------------------------CCU---------------------ACGUCCCACU 5'	MFE: -28.0 kcal/mol

Supplemental Figure 7. The prediction for miRNA binding sites on lncRNA transcript

(a) The prediction for miR-596 binding sites on lncRNA-KRTAP5-AS1 transcript. **(b)** The prediction for miR-3620-3p binding sites on lncRNA-KRTAP5-AS1 transcript. **(c)** The prediction for miR-3620-3p binding sites on lncRNA-TUBB2A transcript.

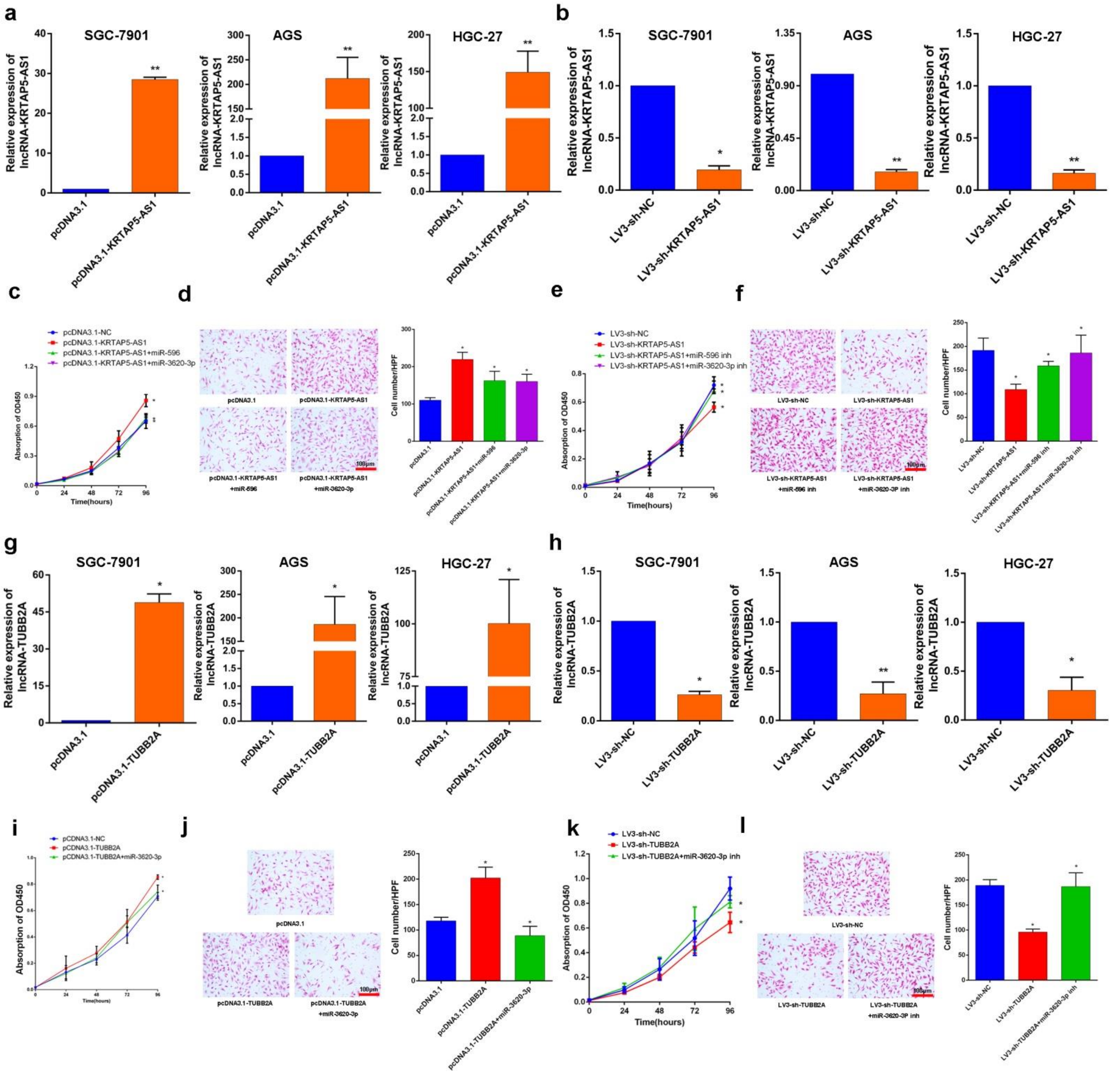

Supplementary Figure 8. The relative expression level of lncRNA-KRTAP5-AS1, lncRNA-TUBB2A

(a) The relative expression level of lncRNA-KRTAP5-AS1 in lncRNA-KRTAP5-AS1 overexpressing GC cell lines. **(b)** The relative expression level of lncRNA-KRTAP5-AS1 in lncRNA-KRTAP5-AS1 knockdown GC cell lines. **(c)** Cell proliferation was assessed daily for four days using the CCK-8 assay in lncRNA-KRTAP5-AS1 overexpressing HGC-27 cells. **(d)** Transwell assays were used to evaluate the effect of lncRNA-KRTAP5-AS1 on invasion in lncRNA-KRTAP5-AS1 overexpressing HGC-27 cells. Cells were incubated for 24h and counted under the microscope. **(e)** Cell proliferation was assessed daily for four days using the CCK-8 assay in lncRNA-KRTAP5-AS1 knockdown HGC-27 cells. **(f)** Transwell assays were used to evaluate the effect of lncRNA-KRTAP5-AS1 on invasion in lncRNA-KRTAP5-AS1 knockdown HGC-27

cells. Cells were incubated for 24h and counted under the microscope. **(g)** The relative expression level of lncRNA-TUBB2A in lncRNA-TUBB2A overexpressing GC cell lines. **(h)** The relative expression level of lncRNA-TUBB2A in lncRNA-TUBB2A knockdown GC cell lines. **(i)** Cell proliferation was assessed daily for four days using the CCK-8 assay in lncRNA-TUBB2A overexpressing HGC-27 cells. **(j)** Transwell assays were used to evaluate the effect of lncRNA-TUBB2A on invasion in lncRNA-TUBB2A overexpressing HGC-27 cells. Cells were incubated for 24h and counted under the microscope. **(k)** Cell proliferation was assessed daily for four days using the CCK-8 assay in lncRNA-TUBB2A knockdown HGC-27 cells. **(l)** Transwell assays were used to evaluate the effect of lncRNA-TUBB2A on invasion in lncRNA-TUBB2A knockdown HGC-27 cells. Cells were incubated for 24h and counted under the microscope. For all microscopic images, Original magnification×200, Scale bars=100μm. Data are shown as mean±s.d., n=3. The data statistical significance is assessed by Student's t-test. *represents $P<0.05$, **represents $P<0.01$.

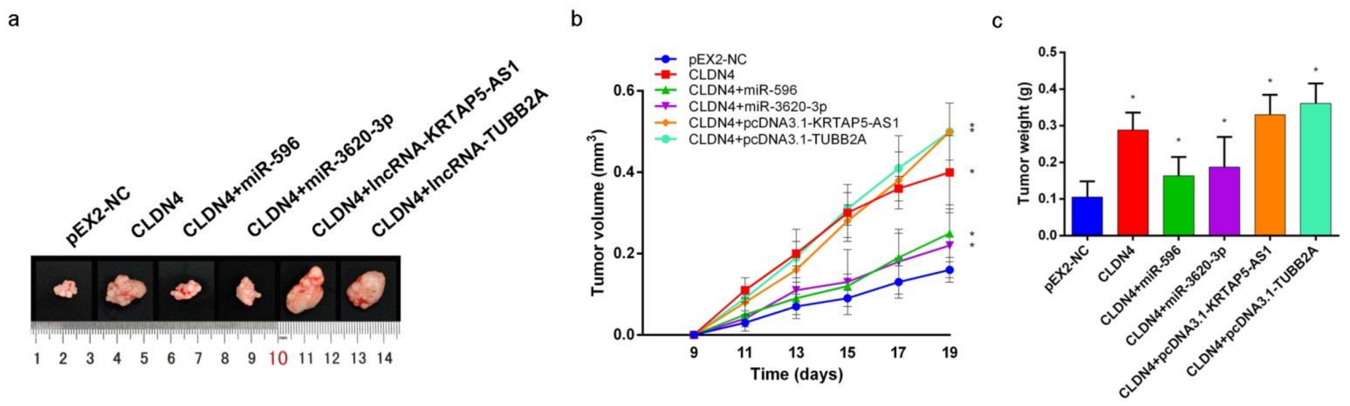

Supplementary Figure 9. CLDN4 promotes proliferation *in vivo*, which can be inhibited by miR-596, miR-3620-3p and enhanced by lncRNA-KRTAP5-AS1, lncRNA-TUBB2A

(**a**) Images of the tumors lumps of subcutaneous xenograft mice. Mice were subcutaneously injected with CLDN4 overexpressing HGC-27 cells, CLDN4 overexpressing cells transfected with mimics of miR-596, miR-3620-3p, pcDNA3.1-KRAP5-AS1 and pcDNA3.1-TUBB2A. Mice were scarified at the 19th day after injection and each tumor lump was removed from the body. (**b**) The tumor growth curves of *in vivo* tumor volumes. Data are mean ± s.d. of the tumor volumes, n=7, *represents P<0.05. (**c**) The mean tumor weight of each group. Data are mean ± s.d. of the tumor volumes, n=7. The data statistical significance is assessed by Student's t-test. *represents P<0.05, ** represents P<0.01.

a

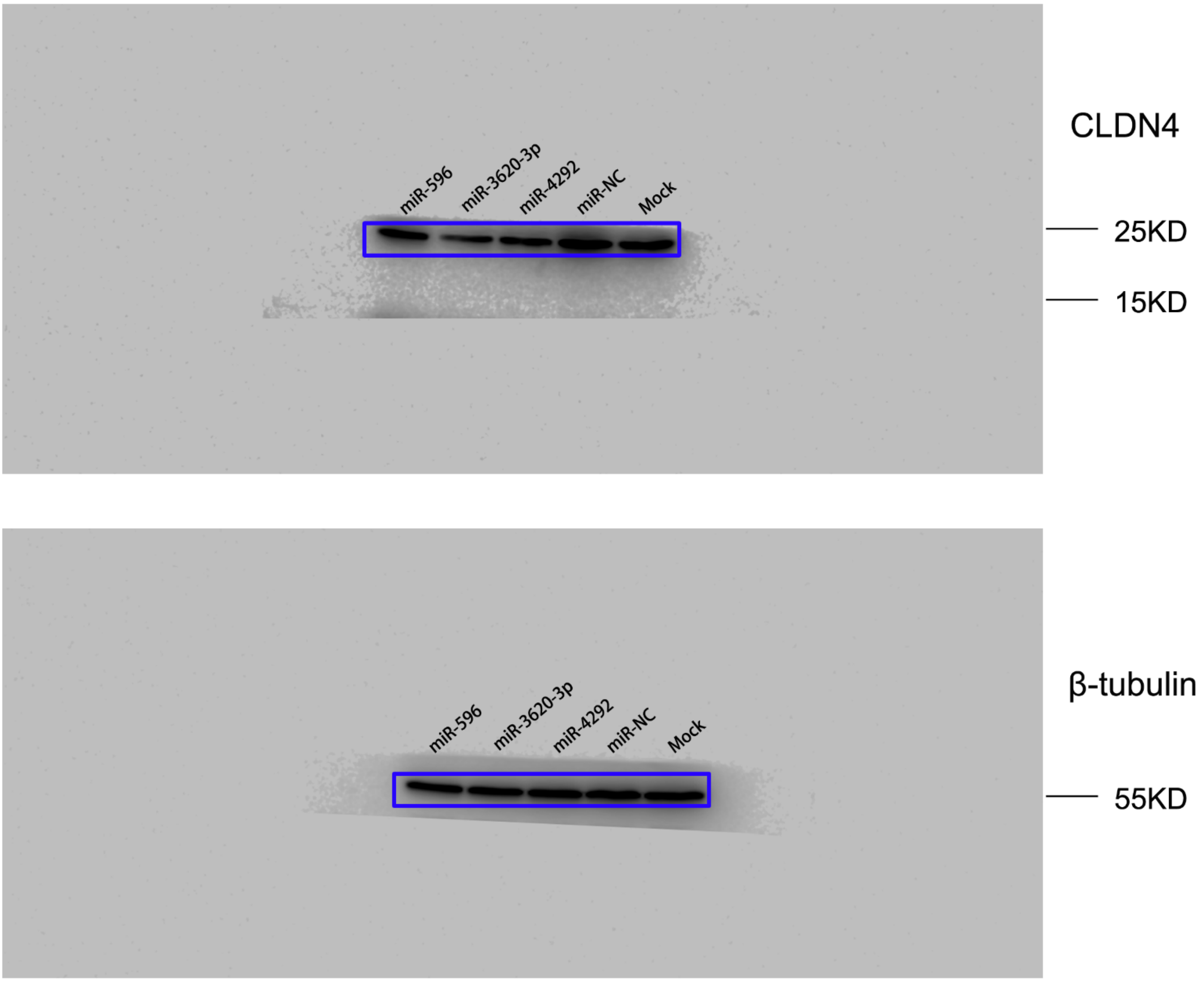
CLDN4
miR-596
miR-3620-3p
miR-4292
miR-NC
Mock
25KD
15KD
β-tubulin
miR-596
miR-3620-3p
miR-4292
miR-NC
Mock
55KD

b

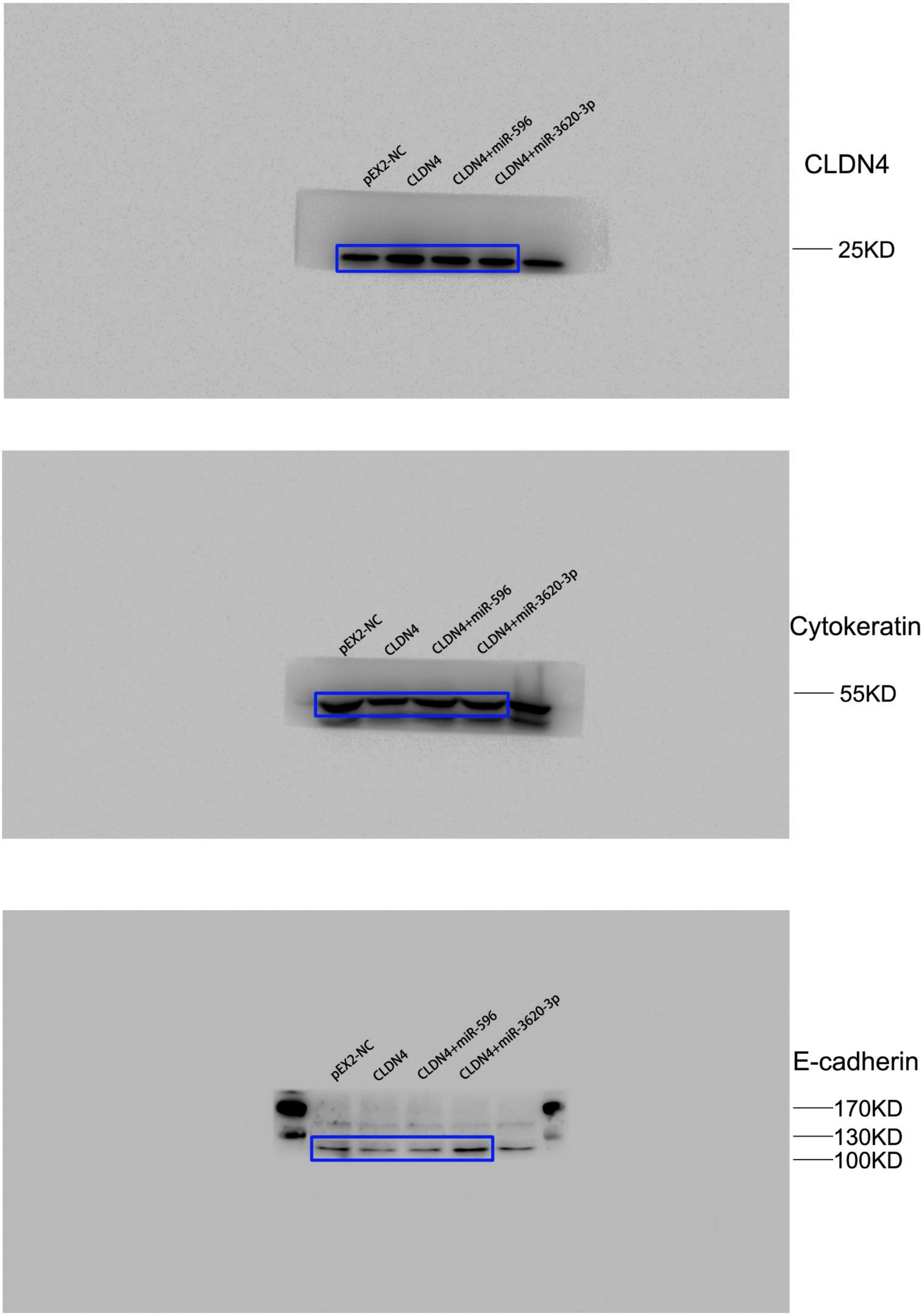
pEX2-NC
CLDN4
CLDN4+miR-596
CLDN4+miR-3620-3p
CLDN4
25KD
pEX2-NC
CLDN4
CLDN4+miR-596
CLDN4+miR-3620-3p
Cytokeratin
55KD
pEX2-NC
CLDN4
CLDN4+miR-596
CLDN4+miR-3620-3p
E-cadherin
170KD
130KD
100KD

c

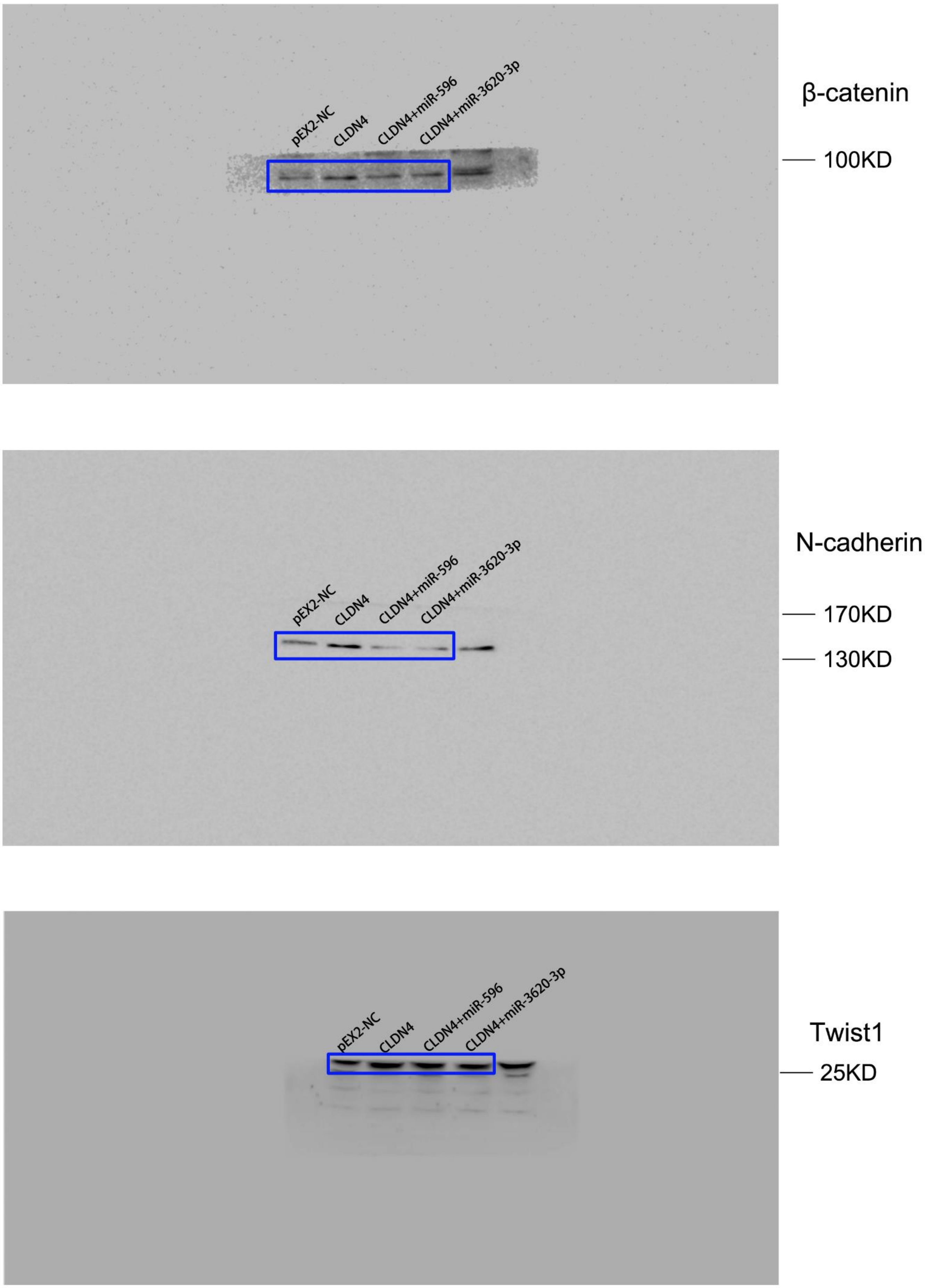
pEX2-NC
CLDN4
CLDN4+miR-596
CLDN4+miR-3620-3p
β-catenin
100KD
pEX2-NC
CLDN4
CLDN4+miR-596
CLDN4+miR-3620-3p
N-cadherin
170KD
130KD
pEX2-NC
CLDN4
CLDN4+miR-596
CLDN4+miR-3620-3p
Twist1
25KD

d

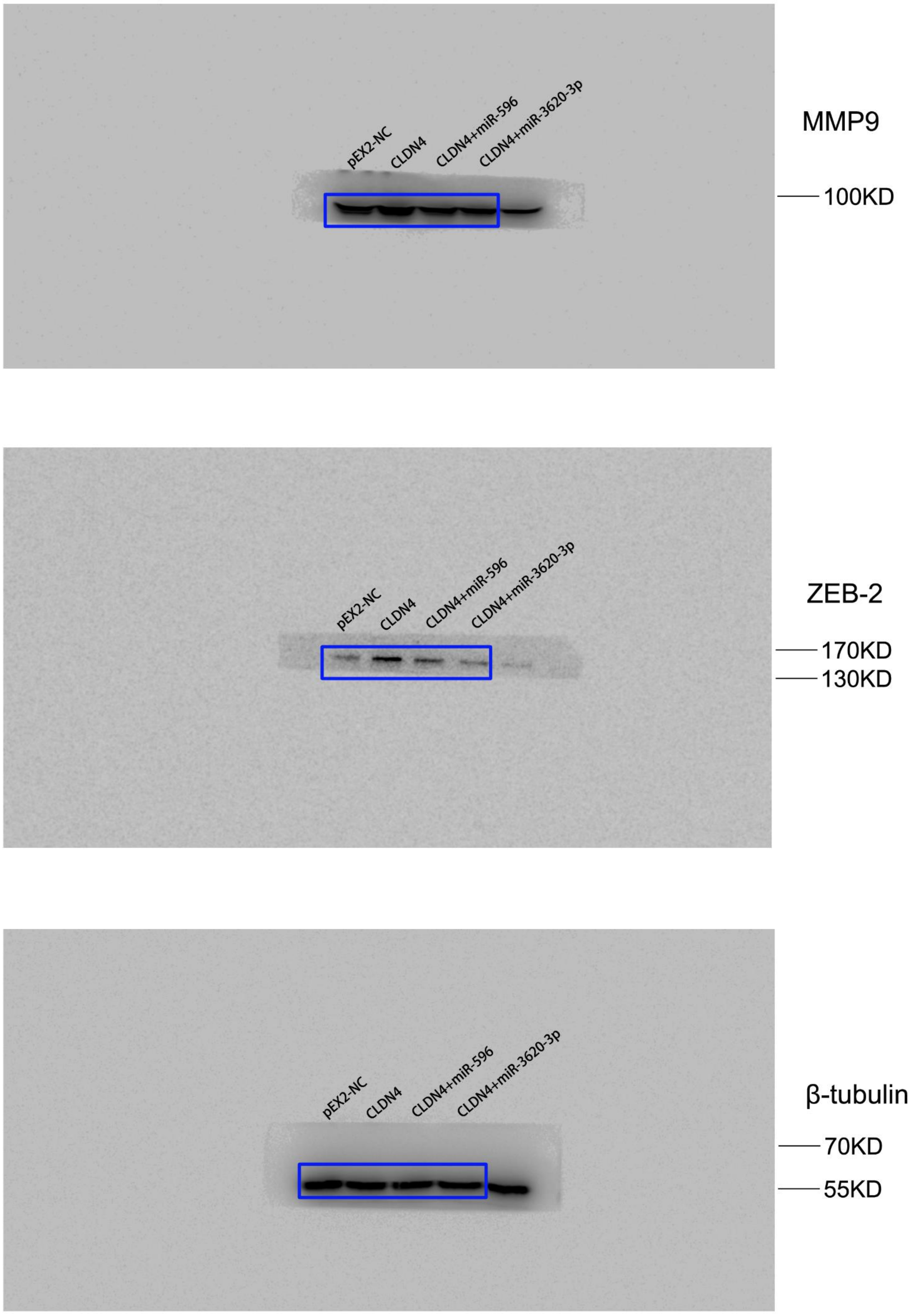
pEX2-NC
CLDN4
CLDN4+miR-596
CLDN4+miR-3620-3p
MMP9
100KD
pEX2-NC
CLDN4
CLDN4+miR-596
CLDN4+miR-3620-3p
ZEB-2
170KD
130KD
pEX2-NC
CLDN4
CLDN4+miR-596
CLDN4+miR-3620-3p
β-tubulin
70KD
55KD

e

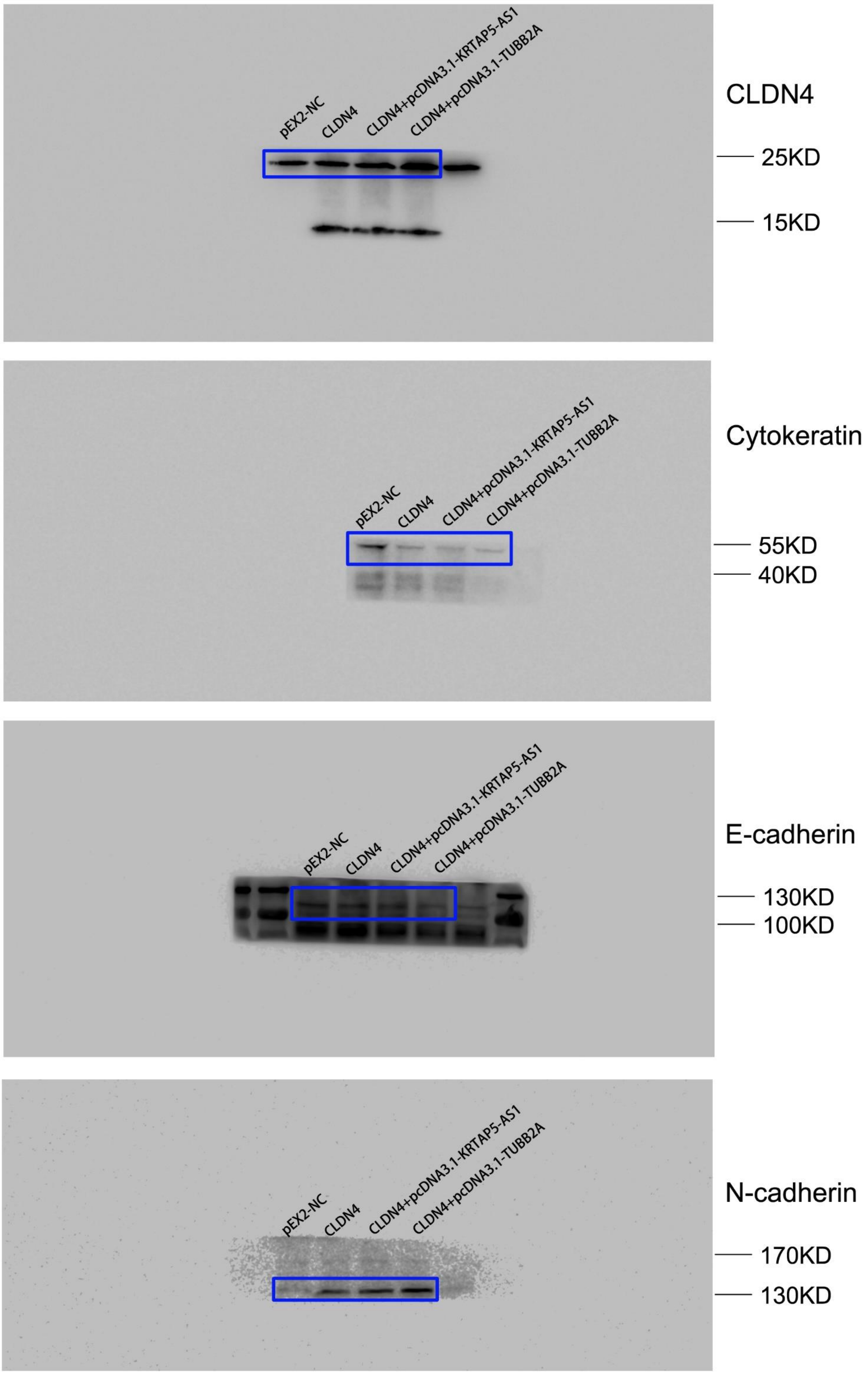
pEX2-NC
CLDN4
CLDN4+pcDNA3.1-KRTAP5-AS1
CLDN4+pcDNA3.1-TUBB2A
CLDN4
25KD
15KD
Cytokeratin
55KD
40KD
E-cadherin
130KD
100KD
N-cadherin
170KD
130KD

f

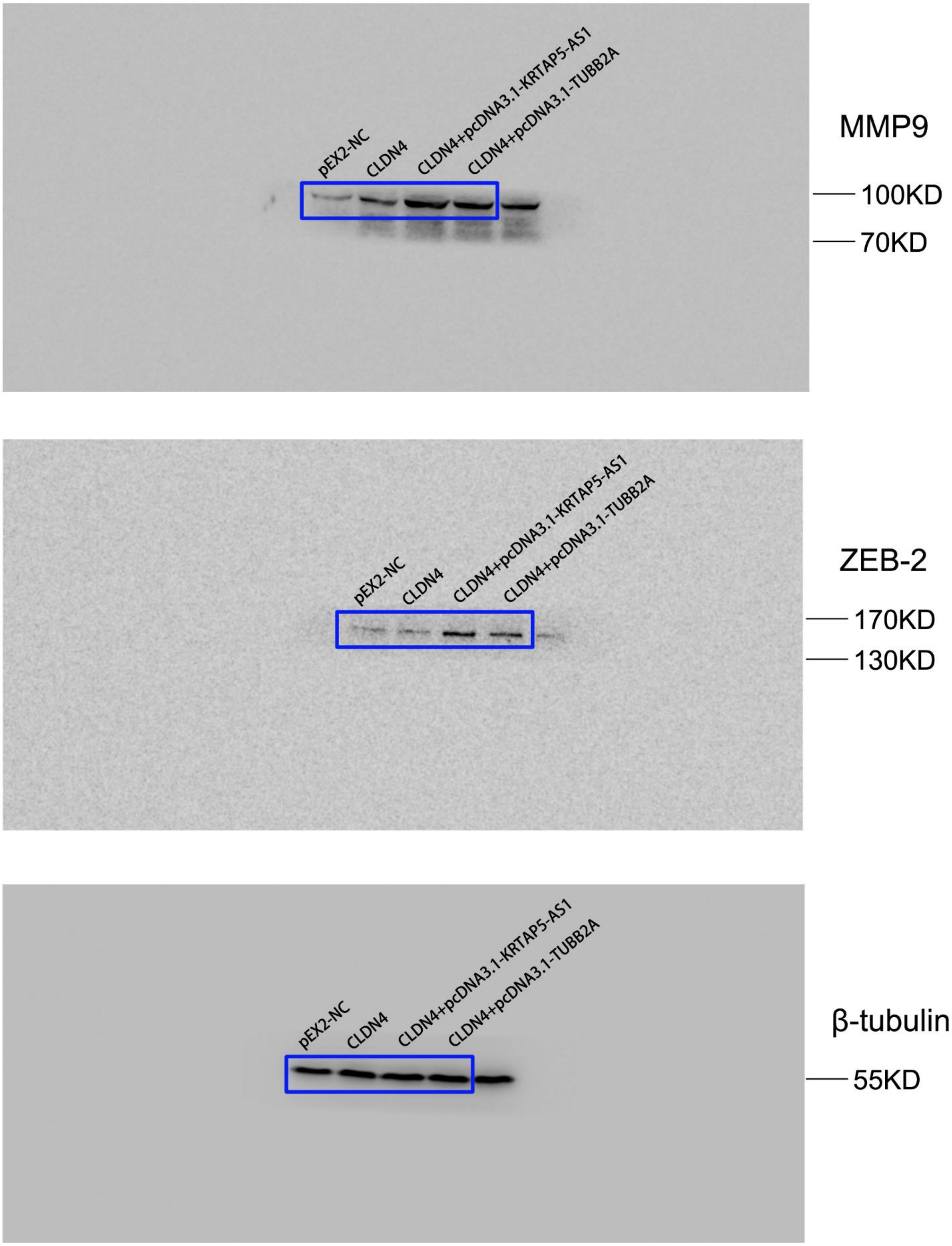

Supplementary Figure 10 Full gel immunoblots for main text figures

(**a**) Full gel immunoblots for Fig. 1e. (**b-d**) Full gel immunoblots for Fig. 1g and Fig. 3c. (**e-f**) Full gel immunoblots for Fig. 6c and Fig. 6g.

Supplementary Table 1. Clinical information of 6 gastric cancer samples used for microarray

Specimen	Age	Gender	Race	Borrmann type	Differentiation	T category	N category	M category	TNM stage	Therapy before Surgery
NO.1	54	M	Asian	III	Poor	T4a	N0	M0	II B	None
NO.2	69	M	Asian	III	Well	T3	N0	M0	II A	None
NO.3	69	M	Asian	III	Poor	T4a	N0	M0	II B	None
NO.4	68	F	Asian	III	Moderate-Poor	T2	N1	M0	II A	None
NO.5	79	F	Asian	II	Moderate-Poor	T4a	N1	M0	III A	None
NO.6	62	M	Asian	I	Poor	T2	N2	M0	II B	None

Abbreviation, F: Female; M: Male.

Supplementary Table 2. Univariate and multivariate survival analyses in 104 gastric cancer patients

	Univariate		Multivariate	
**Variable**	**HR (95% CI)**	**P**	**HR (95% CI)**	**P**
Gender		0.445		
Female	1			
Male	0.816 (0.485-1.375)			
Age (y)		0.149		
<61	1			
≥61	1.389 (0.844-2.286)			
Tumor size (cm)		0.074		
<5	1			
≥5	1.619 (0.954-2.745)			
Borrmann type		0.018		0.071
I-II*	1		1	
III-IV	3.396 (1.231-9.365)		2.620 (0.919-7.467)	
Differentiation		0.065		
Well - moderate	1			
Poor	1.729 (0.967-3.094)			
pT category		0.039		0.214
T1-T2	1		1	
T3-T4	2.189 (1.040-4.611)		1.630 (0.755-3.518)	
pN category		0.007		0.009
N0	1		1	
N1-N3	2.554 (1.296-5.030)		2.520 (1.262-5.034)	
TNM stage		0.001		
I-II	1			
III	2.708 (1.529-4.797)			
CLDN4		0.001		<0.001
Low	1		1	
High	2.350 (1.394-3.960)		2.634 (1.536-4.517)	

Abbreviation, HR: Hazard ratio.

*including 7 cases of early stage.

Supplementary Table 3. Primary antibodies for western blotting

Name	Catalog number	Dilution	Manufacture
E-cadherin	sc-8426	1:200	Santa Cruz Biotechnology, Santa Cruz, CA, USA
Cytokeratin	ab53280	1:2000	Abcam, Shanghai, China
Laminin	ab44941	1:200	Abcam, Shanghai, China
ZO-1	D7D12	1:1000	Cell Signaling Technology, Danvers, MA, USA
N-cadherin	13116s	1:1000	Cell Signaling Technology, Danvers, MA, USA
β-catenin	sc-7963	1:200	Santa Cruz Biotechnology, Santa Cruz, CA, USA
SNAI1	3879s	1:1000	Cell Signaling Technology, Danvers, MA, USA
ZEB-2	sc-271984	1:200	Santa Cruz Biotechnology, Santa Cruz, CA, USA
Twist1	ab50518	1:500	Abcam, Shanghai, China
MMP-9	WL01580	1:800	Wanleibio, Shenyang, China
Claudin4	ab53156	1:1000	Abcam, Shanghai, China
β-tubulin	T7816	1:2000	Sigma–Aldrich, St. Louis, MO, USA

Supplementary Table 4. ShRNAs sequences

Name	Sequence
shRNA-CLDN4	5’-CAAGGACACTAATGAGCCTGG-3’
shRNA-TUBB2A	5’-CGTGCATCCTTAGTGAACTTC-3’
shRNA-KRTAP5-AS1	5’-GTCCCTTTCTTGTGACTGTTT-3’
shRNA-NC	5’-TTTCTCCGAACGTGTCACGTTTC-3’

Supplementary Table 5. RNA oligoribonucleotides sequences

Name	Sequence
miR-596 forward	5'-AAGCCUGCCCGGCUCCUCGGG-3'
miR-596 reverse	5'-CGAGGAGCCGGGCAGGCUUUU-3'
miR-3620-3p forward	5'-UCACCCUGCAUCCCGCACCCAG-3'
miR-3620-3p reverse	5'-GGGUGCGGGAUGCAGGGUGAUU-3'
miR-4292 forward<br>miR-4292 reverse	5'-CCCCUGGGCCGGCCUUGG-3'<br>5'-CCAAGGCCGGCCCAGGUU-3'
miR-596-mut reverse	5'-UACCGUCCCCGGCUCCUCGGG-3'
miR-596-mut reverse	5'-CGAGGAGCCGGGGACGGUAUU-3'
miR-3620-3p-mut forward	5'-ACUCGCAGCAUCCCGCACCCAG-3'
miR-3620-3p-mut reverse	5'-GGGUGCGGGAUGCUGCGAGUUU-3'
miRNA NC forward	5'-UUCUCCGAACGUGUCACGUTT-3'
miRNA NC reverse	5'-ACGUGACACGUUCGGAGAATT-3'
miR-596 inhibitor	5'-CCCGAGGAGCCGGGCAGGCUU-3'
miR-3620-3p inhibitor	5'-CUGGGUGCGGGAUGCAGGGUGA-3'
miRNA inhibitor NC	5'-CAGUACUUUUGUGUAGUACAA-3'

Supplementary Methods

The process of the pathway analysis, key genes selection, network analysis, and mRNA-ceRNA analysis

i) Six pairs of GC tissues and non-tumorous adjacent tissues were analyzed via microarray using the Human LncRNA + mRNA Array v3.0 together with the miRCURY LNATM microRNA Array.

ii) Using a Student's t-test, we identified 4421 differentially expressed lncRNAs and 3369 differentially expressed mRNAs, which were up-regulated or down-regulated more than 2 fold (Supplementary data 2, 3).

iii) Based on the KEGG database, we performed pathway analysis for differentially expressed mRNAs. We chose the cut-off based on p-val and all pathways with a p-val less than 0.05 were proceeded with a deeper analysis. We identified 22 pathways based on the up-regulated genes and 39 pathways based on the down-regulated genes.

iv) Considering the p-val and our previous work, 4 pathways were filtered from 61 pathways. Cell adhesion, pathway in cancer, and cell cycle were among the top candidates and related to cancer development. Moreover, we selected the tight junction pathway because our previous work demonstrated aberrant expression of CLDN4, a component of the tight junction pathway, in gastric cancer and precursor lesions[1]. Also, using meta-analysis, we previously found that CLDN4 expression was associated with increasing pT category, tumor size, and lymph node metastasis in patients with gastric cancer[2]. Therefore, we focused on the tight junction pathway and chose CLDN4 for our key target.

v) After pathways selection, we chose key mRNAs from the differentially expressed mRNA list for each pathway according to previous reports on their relation with gastric cancer and the p-val. For example, there were 20 significantly highly-expressed mRNAs in the tight junction pathway, and 4 of them (CLDN1, CLDN4, CLDN7 and HRAS) were reported to be highly expressed in gastric cancer. Considering that the p-val of HRAS was relatively high (P=0.019), we only selected CLDN1, CLDN4, and CLDN7 to proceed with a deeper analysis. After this selection, the remaining key genes were:

**Pathways**	**Selected key genes**
Cell adhesion	CLDN18; ITGA4; PTPRC
Pathway in cancer	RAC1; VEGFA; MMP1; ERBB2; HRAS; IL8
Tight junction	CLDN4; CLDN7; CLDN1
Cell cycle	CCNB1; CCNE1; CDK1; CDK7; SFN; MYC

vi) We then predicted the miRNAs which could regulate the key mRNAs above. There were three criteria. The expression of a miRNA was significantly different between cancer and non-tumorous adjacent tissues; the miRNA was up-regulated if the candidate target mRNA was down-regulated, or down-regulated if the candidate target mRNA was up-regulated; and context score<0 and context+ score<0[3-5].

vii) After removing the mRNAs with no apparent miRNA regulation, the list of miRNA-mRNA interactions was as follows:

**Pathways**	**MiRNA-mRNA pairs**
Cell adhesion	hsa-miR-3689b-3p & CLDN18;
	hsa-miR-3689c & CLDN18
	hsa-miR-3121-5p & ITGA4
Pathway in cancer	hsa-miR-22-5p & MMP1
	hsa-miR-4292 & HRAS
	hsa-miR-136-5p & RAC1
	hsa-miR-1470 & VEGFA
	hsa-miR-125b-5p & VEGFA
Tight junction	hsa-miR-596 & CLDN4
	hsa-miR-3620-3p & CLDN4
	hsa-miR-4292 & CLDN4
	hsa-miR-624-5p & CLDN1
	hsa-miR-596 & CLDN7
	hsa-miR-3620-3p & CLDN7
Cell cycle	hsa-miR-628-3p & CDK1
	hsa-miR-22-5p & CDK1

viii) The ceRNAs were predicted for each miRNA-mRNA pair using the MuTaMe Score[6], with the cut-off set as MuTaMe Score>0.005. All 4421 differentially expressed lncRNAs and 3369 differentially expressed mRNAs were entered into this ceRNA prediction as candidates (Supplementary Data 2, 3). The interactions were graphed using the Cytoscape software (v2.8.1) (Fig.1b).

The process of the RNA-sequencing and data analysis

Total RNA from each sample was quantified using a NanoDrop ND-1000 instrument. 1-2 μg of total RNA was selected for each sample to construct a sequencing library. Total RNA was enriched by oligo(dT) magnetic beads using the NEBNext® Poly (A) mRNA Magnetic Isolation Module. After processing, the RNA was used to construct a sequencing library using the KAPA Stranded RNA-Seq Library Prep Kit (Illumina), which included procedures for RNA fragmentation, random hexamer-primed first strand cDNA synthesis, dUTP-based second strand cDNA synthesis, end-repairing, A-tailing, adaptor ligation, and library PCR amplification. The completed libraries were qualified on an Agilent 2100 Bioanalyzer and quantified by the absolute quantification qPCR method. To sequence the libraries on the Illumina HiSeq 4000 instrument, the barcoded libraries were mixed, denatured to single stranded DNA in NaOH, captured on an Illumina flow cell, amplified in situ, and sequenced for 150 cycles for both ends on the Illumina HiSeq 4000 instrument.

Image analysis and base calling were performed using Solexa pipeline v1.8 (Off-Line Base Caller software, v1.8). Sequence quality was examined using FastQC software[7]. The trimmed reads (trimmed 5', 3'-adaptor bases using cutadapt[8]) were aligned to the reference genome (hg19) using Hisat2 software (v2.0.4)[9]. The transcript abundances for each sample were estimated with StringTie (v1.2.3)[10], and the FPKM[11] value for gene and transcript levels were calculated with R package Ballgown (v2.6.0)[12,13]. The differential expression analysis of FPKM was based on Significance B using Perseus software (1.5.3.2)[14-16]. The false-discovery rate (FDR) adjusted P values for multiple testing were calculated with the Benjamini-Hochberg method[17].

Supplementary References

1. Zhu, J.L. et al. Clinicopathological significance of claudin-4 in gastric carcinoma. *World J Surg Oncol* **11**, 150 (2013).
2. Chen, X. et al. Clinicopathological significance of claudin 4 expression in gastric carcinoma: a systematic review and meta-analysis. *Onco Targets Ther* **9**, 3205-12 (2016).
3. Friedman, R.C., Farh, K.K., Burge, C.B. & Bartel, D.P. Most mammalian mRNAs are conserved targets of microRNAs. *Genome Res* **19**, 92-105 (2009).
4. Garcia, D.M. et al. Weak seed-pairing stability and high target-site abundance decrease the proficiency of lsy-6 and other microRNAs. *Nat Struct Mol Biol* **18**, 1139-46 (2011).
5. Grimson, A. et al. MicroRNA targeting specificity in mammals: determinants beyond seed pairing. *Mol Cell* **27**, 91-105 (2007).
6. Tay, Y. et al. Coding-independent regulation of the tumor suppressor PTEN by competing endogenous mRNAs. *Cell* **147**, 344-57 (2011).
7. FastQC. http://www.bioinformatics.babraham.ac.uk/projects/fastqc/.
8. Martin M. Cutadapt removes adapter sequences from high-throughput sequencing reads. EMBnet J **17**, 10-12 (2011).
9. Kim, D., Langmead, B. & Salzberg, S.L. HISAT: a fast spliced aligner with low memory requirements. Nat Methods **12**, 357-60 (2015).
10. Pertea, M. et al. StringTie enables improved reconstruction of a transcriptome from RNA-seq reads. Nat Biotechnol **33**, 290-5 (2015).
11. Mortazavi, A., Williams, B.A., McCue, K., Schaeffer, L. & Wold, B. Mapping and quantifying mammalian transcriptomes by RNA-Seq. Nat Methods **5**, 621-8 (2008).
12. Frazee, A.C., Pertea, G. & Jaffe, A.E. Ballgown bridges the gap between transcriptome assembly and expression analysis. Nat Biotechnol **33**, 243-6 (2015).

13. Fu, J., Frazee, A.C., Collado-Torres, L., Jaffe, A.E., Leek, J.T. ballgown: Flexible, isoform-level differential expression analysis. R package version 2.6.0. https://bioconductor. org/packages/ release/bioc/ html/ballgown.html (2015).
14. Leek, J.T., Tyanova, S., Temu, T. & Sinitcyn, P. The Perseus computational platform for comprehensive analysis of (prote)omics data. Nat Methods **13**, 731-40 (2016).
15. Geiger, T., Mann, M., Cox, J., Cox, J. & Mann, M. 1D and 2D annotation enrichment: a statistical method integrating quantitative proteomics with complementary high-throughput data. BMC bioinformatics **13 Suppl 16**, S12 (2012).
16. Cox, J. & Mann, M. MaxQuant enables high peptide identification rates, individualized p.p.b.-range mass accuracies and proteome-wide protein quantification. Nat Biotechnol **26**, 1367-72 (2008).
17. Benjamini, Y. & Hochberg, Y. Controlling The False Discovery Rate - A Practical And Powerful Approach To Multiple Testing. Journal of the Royal Statistical Society **57**, 289-300 (1995).
